# The protocadherin 17 gene affects cognition, personality, amygdala structure and function, synapse development and risk of major mood disorders

**DOI:** 10.1038/mp.2016.231

**Published:** 2017-01-10

**Authors:** H Chang, N Hoshina, C Zhang, Y Ma, H Cao, Y Wang, D-d Wu, S E Bergen, M Landén, C M Hultman, M Preisig, Z Kutalik, E Castelao, M Grigoroiu-Serbanescu, A J Forstner, J Strohmaier, J Hecker, T G Schulze, B Müller-Myhsok, A Reif, P B Mitchell, N G Martin, P R Schofield, S Cichon, M M Nöthen, The Swedish Bipolar Study Group, Lena Backlund, Louise Frisén, Catharina Lavebratt, Martin Schalling, Urban Ösby, MooDS Bipolar Consortium, Thomas W Mühleisen, Markus Leber, Franziska Degenhardt, Jens Treutlein, Manuel Mattheisen, Anna Maaser, Sandra Meier, Stefan Herms, Per Hoffmann, André Lacour, Stephanie H Witt, Fabian Streit, Susanne Lucae, Wolfgang Maier, Markus Schwarz, Helmut Vedder, Jutta Kammerer-Ciernioch, Andrea Pfennig, Michael Bauer, Martin Hautzinger, Adam Wright, Janice M Fullerton, Grant W Montgomery, Sarah E Medland, Scott D Gordon, Tim Becker, Johannes Schumacher, Peter Propping, H Walter, S Erk, A Heinz, N Amin, C M van Duijn, A Meyer-Lindenberg, H Tost, X Xiao, T Yamamoto, M Rietschel, M Li

**Affiliations:** 1 Key Laboratory of Animal Models and Human Disease Mechanisms of the Chinese Academy of Sciences and Yunnan Province, Kunming Institute of Zoology, Kunming, Yunnan, China; 2 Cell Signal Unit, Okinawa Institute of Science and Technology Graduate University, Okinawa, Japan; 3 Department of Neurology, F.M. Kirby Neurobiology Center, Boston Children's Hospital, Harvard Medical School, Boston, MA, USA; 4 Division of Mood Disorders, Shanghai Mental Health Center, Shanghai Jiao Tong University School of Medicine, Shanghai, China; 5 State Key Laboratory of Cognitive Neuroscience and Learning, IDG/McGovern Institute for Brain Research, Beijing Normal University, Beijing, China; 6 Department of Psychiatry and Psychotherapy, Central Institute of Mental Health, Medical Faculty Mannheim, University of Heidelberg, Mannheim, Germany; 7 State Key Laboratory of Genetic Resources and Evolution, Kunming Institute of Zoology, Chinese Academy of Sciences, Kunming, Yunnan, China; 8 Department of Medical Epidemiology and Biostatistics, Karolinska Institutet, Stockholm, Sweden; 9 Stanley Center for Psychiatric Research, Broad Institute of MIT and Harvard, Cambridge, MA, USA; 10 Section of Psychiatry and Neurochemistry, Sahlgrenska Academy at Gothenburg University, Gothenburg, Sweden; 11 Department of Psychiatry, Centre Hospitalier Universitaire Vaudois, Prilly, Switzerland; 12 Institute of Social and Preventive Medicine, Centre Hospitalier Universitaire Vaudois, Lausanne, Switzerland; 13 Swiss Institute of Bioinformatics, Lausanne, Switzerland; 14 Biometric Psychiatric Genetics Research Unit, Alexandru Obregia Clinical Psychiatric Hospital, Bucharest, Romania; 15 Institute of Human Genetics, University of Bonn, Bonn, Germany; 16 Department of Genomics, Life and Brain Center, University of Bonn, Bonn, Germany; 17 Department of Genetic Epidemiology in Psychiatry, Central Institute of Mental Health, Medical Faculty Mannheim, University of Heidelberg, Mannheim, Germany; 18 Institute of Genomic Mathematics, University of Bonn, Bonn, Germany; 19 Institute of Psychiatric Phenomics and Genomics, Ludwig-Maximilians-University Munich, Munich, Germany; 20 Max Planck Institute of Psychiatry, Munich, Germany; 21 Munich Cluster for Systems Neurology (SyNergy), Munich, Germany; 22 University of Liverpool, Institute of Translational Medicine, Liverpool, UK; 23 Department of Psychiatry, Psychosomatic Medicine and Psychotherapy, University Hospital Frankfurt, Frankfurt, Germany; 24 School of Psychiatry, University of New South Wales, Sydney, NSW, Australia; 25 Black Dog Institute, Sydney, NSW, Australia; 26 QIMR Berghofer Medical Research Institute, Brisbane, QLD, Australia; 27 Neuroscience Research Australia, Sydney, NSW, Australia; 28 School of Medical Sciences, University of New South Wales, Sydney, NSW, Australia; 29 Division of Medical Genetics, University Hospital Basel and Department of Biomedicine, University of Basel, Basel, Switzerland; 30 Institute of Neuroscience and Medicine (INM-1), Structural and Functional Organization of the Brain, Genomic Imaging, Research Centre Jülich, Jülich, Germany; 31 Department of Psychiatry and Psychotherapy, Universitätsmedizin Charité, Berlin, Germany; 32 Department of Epidemiology, Erasmus University Medical Center, Rotterdam, The Netherlands

## Abstract

Major mood disorders, which primarily include bipolar disorder and major depressive disorder, are the leading cause of disability worldwide and pose a major challenge in identifying robust risk genes. Here, we present data from independent large-scale clinical data sets (including 29 557 cases and 32 056 controls) revealing brain expressed protocadherin 17 (*PCDH17*) as a susceptibility gene for major mood disorders. Single-nucleotide polymorphisms (SNPs) spanning the *PCDH17* region are significantly associated with major mood disorders; subjects carrying the risk allele showed impaired cognitive abilities, increased vulnerable personality features, decreased amygdala volume and altered amygdala function as compared with non-carriers. The risk allele predicted higher transcriptional levels of *PCDH17* mRNA in postmortem brain samples, which is consistent with increased gene expression in patients with bipolar disorder compared with healthy subjects. Further, overexpression of *PCDH17* in primary cortical neurons revealed significantly decreased spine density and abnormal dendritic morphology compared with control groups, which again is consistent with the clinical observations of reduced numbers of dendritic spines in the brains of patients with major mood disorders. Given that synaptic spines are dynamic structures which regulate neuronal plasticity and have crucial roles in myriad brain functions, this study reveals a potential underlying biological mechanism of a novel risk gene for major mood disorders involved in synaptic function and related intermediate phenotypes.

## INTRODUCTION

A major challenge in modern medicine is to understand the molecular and cellular mechanisms underlying common mental illnesses such as the major mood disorders, which primarily include bipolar disorder (BPD) and major depressive disorder (MDD) with a combined lifetime prevalence of up to 20% and a leading cause of morbidity worldwide.^1^ Clinical, epidemiological and genetic findings have suggested shared risk factors between BPD and MDD.^2^ Despite considerable evidence of heritability,^2^ the neurobiology of major mood disorders remains poorly understood due to the lack of biomarkers, phenotypic uncertainties, and heterogeneity of precipitating factors. However, accumulating basic and clinical studies point to aberrant structures and dysfunction of brain regions that engage in emotional and cognitive processes, such as prefrontal cortex, hippocampus and amygdala. Dysregulated neuronal synapses in these brain areas,^3, 4^ and the effect of antidepressive medication on these brain regions^5^ also suggest their involvement in the neurobiological mechanism of major mood disorders. Therefore, gene identification for complex diseases such as major mood disorders will require demonstration that risk variants affect the key aspects of the biology of the illness.^6^ Indeed, this logic has proven to be crucial in a number of complex disorders, such as adult onset diabetes, in which multiple genes each account for only a very small share of genetic risk, but show stronger effects on related intermediate phenotypes even in healthy individuals, such as body mass index^7^ or glucose-induced insulin release.^8^

To date, genome-wide association studies (GWAS) have been conducted in several mood disorder samples, identifying several genome-wide significant genes (either across disorders or for single illness), such as *ANK3*, *CACNA1C*, *ODZ4*, *NCAN*, *PBRM1* and *TRANK1*.^9, 10, 11, 12, 13, 14, 15^ It has been increasingly recognized that major mood disorders are polygenic, with numerous alleles each accounting for a very small share of genetic risk in the illness.^16^ Those genome-wide significant genes only explain a small portion of the genetic liability and the sources of missing heritability are still unclear. GWAS is an effective tool to discover the novel risk genes with strong effects, as it entails scanning the genome with hundreds of thousands of genetic variations and employing rigid statistical correction to avoid false positives. This strategy has been successful for several complex disorders with very large samples, such as schizophrenia,^17^ type 2 diabetes^18^ and blood pressure.^19^ However, when the sample sizes are inadequate, the stringent corrections of GWAS preclude the ability to discover genuine risk genes that are of substantial biological interest in spite of only reaching nominal significance. Alternatively, at present, other approaches such as convergent functional genomics,^20^ gene expression profiling,^21^ or candidate gene studies with *a priori* hypotheses^22^ are still necessary to identify potential susceptibility genes for major mood disorders.

The cell adhesion proteins, protocadherins, through their effects on guiding neurons during development, neuronal differentiation and synaptogenesis,^23^ are feasible targets in the pathogenesis of major mood disorders. Among those protocadherin family members, *PCDH17* (protocadherin 17) is expressed by a subset of amygdala neurons,^24^ and *PCDH17* knockout mice exhibit antidepressant–like phenotypes,^25^ implying potential involvement of *PCDH17* in major mood disorders. Notably, a previous linkage study on mood–incongruent psychotic features showed strong evidence for linkage on human chromosome 13q21-33,^26^ the genomic region where *PCDH17* gene was located (13q21.1). These convergent lines of evidence suggest *PCDH17* is a plausible susceptibility gene for major mood disorders, but direct evidence of association is still absent.

Here, we conduct a meta-analysis of independent clinical samples including a total of 29 557 cases and 32 056 controls and we report a novel risk candidate gene *PCDH17* for major mood disorders. Single-nucleotide polymorphisms (SNPs) spanning the *PCDH17* region were found to be associated with mood disorders across multiple independent samples. We describe its association with several biological intermediate phenotypes such as cognition, emotion, and amygdala structures and functions. We show that the risk alleles predict higher *PCDH17* mRNA levels in postmortem brains, consistent with its increased expression in patients compared with healthy controls. Elevated expression of *PCDH17* in primary neuronal cultures revealed decreased spine density and aberrant dendritic morphology. Such changes in dendritic spines may underlie abnormalities in synaptic function thought to be a fundamental aspect of brain dysfunction in major mood disorders. These convergent results implicate *PCDH17* in the biology of synapses and in the etiology and pathophysiology of major mood disorders, making it a potential new target for the pharmacotherapy of these conditions.

## Materials and methods

All the protocols and methods used in this study were approved by the institutional review board of the Kunming Institute of Zoology, Chinese Academy of Sciences and adhere to all relevant national and international regulations.

### Clinical association samples

In the discovery stage, we performed a meta-analysis using statistics from a BPD GWAS which has been described in Ruderfer *et al.*^27^ and a non-overlapped MDD GWAS which has been described elsewhere.^28^ In brief, the BPD GWAS sample included 10 410 cases and 10 700 controls, it has partial overlap with the Psychiatric Genomics Consortium (PGC) BPD GWAS,^12^ but also includes four additional BPD samples compared with the PGC GWAS.^12^ Standardized semi-structured interviews were used to collect clinical information about lifetime history of psychiatric illness, and operational criteria were applied to make lifetime diagnoses. All cases have experienced pathologically relevant episodes of elevated mood (mania or hypomania) and meet the criteria for BPD within the primary study classification system. Controls were selected from the same geographical and ethnic populations as the cases and had a low probability of having BPD.

The MDD GWAS includes 9227 patients and 7383 controls. Cases were required to have diagnoses of DSM-IV lifetime MDD established using structured diagnostic instruments from direct interviews by trained interviewers or clinician-administered DSM-IV checklists. Most samples ascertained cases from clinical sources, and most controls were randomly selected from the population and screened for lifetime history of MDD. In each GWAS, logistic regression was applied to test the association of clinical diagnosis with SNP dosages under an additive model. Covariates included sample grouping and principal components reflecting ancestry. Detailed descriptions of the samples, data quality, genomic controls and statistical analyses can be found in the original GWAS.^27, 28^

Replication analyses were performed in nine independent BPD or MDD samples that included 9920 patients and 13 973 controls, and no overlap was found with the discovery samples. Detailed information on individual samples—including diagnostic assessment, genotyping and quality control—are shown in the Supplementary Data and Supplementary Table 1. Most of these replication samples were previously reported in earlier large-scale collaborative studies where they were found to be effective in detecting genetic risk variants for BPD.^10, 22, 29^ Each of the original sample subjects were recruited under relevant ethical and legal guidelines for their respective areas, and all provided written informed consents prior to their inclusion in the earlier studies. In brief, the origin and sizes of the replication samples are as follows: (1) Sweden (1415 BPD cases and 1271 controls);^29^ (2) Romania (461 BPD cases and 329 controls);^30^ (3) Germany II (181 BPD cases and 527 controls);^14, 29^ (4) Australia (330 BPD cases and 1811 controls);^14, 22^ (5) USA (58 BPD cases and 145 controls); (6) China-I (198 BPD cases and 135 controls); (7) PsyCoLaus (1585 MDD cases and 2362 controls);^15, 29^ (8) China-II (5303 MDD cases and 5337 controls);^31^ (9) The Netherlands (389 MDD cases and 2056 controls).^32^

### SNP selection, genotyping and statistical analysis

For genotyping in our replication samples, we mainly used the Illumina (San Diego, CA, USA) and Affymetrix platforms (details are shown in Supplementary Data), and the genotyping yield was at least 99% in cases and control subjects of all groups. During statistical analysis, for initial screening in the discovery BPD and MDD samples, the statistics data from a total of 559 SNPs covering 2.0 Mb in 13q21.1 region were obtained from both GWAS samples. We utilized PLINK v1.07 to perform the meta-analysis of the 559 SNPs in two samples. We used odds ratio (OR) and standard error (SE) to estimate heterogeneity between individual samples and to calculate the pooled OR and 95% confidence interval (CI) in the combined samples. To combine the results from individual sample, we calculated the heterogeneity between each samples using the Cochran’s (Q) *χ*^2^-test, which is a weighted sum of the squares of the deviations of individual OR estimates from the overall estimate. In the absence of heterogeneity among individual studies, we used a fixed-effect model to combine the sample and to calculate the pooled OR and the corresponding 95% CIs; otherwise, a random-effect model was applied. The meta-analysis was performed using the classical inverse variance weighted methods. These regional association results of 559 SNPs were plotted using LocusZoom (http://locuszoom.sph.umich.edu/locuszoom/).^33^ During the replication analysis and all combined analysis on rs9537793, ‘*metafor*’ package in R (http://www.R-project.org) was used to perform the meta-analysis using appropriate genetic model. We used a forest plot to graphically present the pooled ORs and the 95% CIs of rs9537793. Each study was represented by a square in the plot, and the weight of each study was also shown. As described in a previous GWAS meta-analysis,^12^ *P-*values for replication samples are reported as one-tailed tests and *P-values* for all combined samples are shown as two-tailed tests. *P*-value <8.94 × 10^−5^ was set as the statistical significance level in the discovery and combined samples; in the replication sample, *P*-value <0.05 was considered significant.

### Cognitive index

We used educational attainment as a ‘proxy phenotype’ for cognitive function. Although it’s not a direct cognitive measure, educational attainment is correlated with cognitive ability (*r*~0.5) and some personality traits related to persistence and self-discipline.^34^ Educational attainment is strongly associated with social outcomes, and there is a well-documented health-education gradient. Estimates suggest that around 40% of the variance in educational attainment is explained by genetic factors.^34^ The harmonized measurements of educational attainment were coded by study-specific measures using the International Standard Classification of Education (1997) scale,^35^ and included a binary variable for college completion (named ‘College’, that is, whether college degree was completed) and a quantitative variable defined as an individual’s years of schooling (named ‘EduYears’, that is, number of years of schooling completed). College may be more comparable across countries, whereas EduYears contains more information about individual differences within countries. Recently, a GWAS on these ‘educational attainment’ phenotypes has been performed in 101 069 European individuals,^34^ and we utilized the statistical results from their GWAS as our first-step analysis. Briefly, educational attainment was measured at an age at which participants were very likely to have completed their education (more than 95% of the sample was at least 30). On average, participants have 13.3 years of schooling, and 23.1% have a college degree. In the second-step analysis,^36^ we used a sample which included increasing number of subjects (*n*=293 723) and has partial overlap with the first-step sample; in this sample, only ‘EduYears’ phenotype was assessed, with the same standard of measurement with the first-step analysis. Detailed information on the samples, genotyping methods and statistical analyses can be found in the original GWAS report.^34, 36^

### Personality traits measurement

Personality can be deemed as a set of characteristics that influence people’s thoughts, feelings and behavior across a variety of settings. Over the last century, scientific consensus has converged on a taxonomic model of personality traits based on five higher-order dimensions of neuroticism, extraversion, openness to experience, agreeableness and conscientiousness, known as the five-factor model.^37^ Neuroticism refers to the tendency to experience diverse and relatively more intense negative emotions, and is commonly defined as emotional instability; it involves the experience of negative emotions such as anxiety, depression, hostility and the vulnerability to stress. Neuroticism is a pervasive risk factor for different psychiatric conditions including mood disorders and personality disorders, and is also associated with entail emotional dysregulation.^38, 39^

In 2015, the Genetics of Personality Consortium (GPC) conducted GWAS on neuroticism^40^ in 63 661 individuals from Europe, United States and Australia. We obtained the statistical results of *PCDH17* risk SNP from this GWAS as our discovery analysis.^40^ In brief to their GWAS, neuroticism scores were harmonized across all 29 discovery cohorts by item response theory analysis and statistics were performed against SNPs using additive linear regression, with sex, age and principal components as covariates. Later in 2016, Okbay *et al.*^41^ performed an expanded analysis (*n*=170 911) by pooling summary statistics from the published study by the GPC^40^ (*n*=63 661) with results from a new analysis of UK Biobank data^42^ (UKB, *n*=107 245). In the UKB cohort, the measure was the respondent’s score on a 12-item version of the Eysenck Personality Inventory Neuroticism.

### Subcortical structure testing

Subcortical brain regions form circuits with cortical areas to learning, memory^43^ and motivation,^44^ and altered circuits can lead to abnormal behavior and disease.^45^ To investigate how common genetic variants affect the structure of these brain regions, ENIGMA2 consortium conducted GWASs on the volumes of several subcortical regions derived from magnetic resonance images (MRI).^46^

We focused on two phenotypes (amygdala volume and hippocampal volume) closely relevant to risk of mood disorders, and obtained the statistical results from the ENIGMA2 GWAS discovery sample.^46^ In short, the discovery sample includes 13 171 European subjects, the subcortical brain measures (amygdala and hippocampus) were delineated in the brain using well-validated, freely available brain segmentation software packages: FIRST, part of the FMRIB Software Library (FSL), or FreeSurfer. The standardized protocols for image analysis and quality assurance are openly available online (http://enigma.ini.usc.edu/protocols/imaging-protocols/). For each SNP, the additive dosage value was regressed against the trait of interest separately using a multiple linear regression framework controlling for age, age,^2^ sex, four MDS components, ICV and diagnosis (when applicable). For studies with data collected from several centers or scanners, dummy-coded covariates were also included in the model. Detailed information on the samples, imaging procedures and genotyping methods can be found in the original GWAS.^46^

### Functional MRI analysis

#### Imaging Subjects

Functional magnetic resonance images were obtained from healthy German participants (*N*=297) of European ancestry, as part of a tricentric study on the neurogenetic mechanisms of psychiatric disease (the MooDS cohort).^47, 48, 49^ The subjects were recruited from the communities in Mannheim, Bonn and Berlin (mean age 33.77±9.81 years, 134 males and 163 females). Exclusion criteria included a lifetime history of significant general medical, psychiatric or neurological illness, prior drug or alcohol abuse, head trauma, and the presence of a first-degree relative with mental illness. This particular experiment was approved by the ethics committees of the Universities of Bonn, Heidelberg and Berlin. All subjects provided written informed consent to participate in the study.

#### Genotyping

rs9537793 genotyping was performed using Illumina Human 610-Quad and Illumina Human 660 W-Quad arrays (Illumina). The allele frequencies for the SNPs were in the Hardy–Weinberg equilibrium (77 AA, 146 AG, 74 GG, *P*=0.77). Age, handedness, sex, site and level of education did not significantly differ between genotype groups (see Supplementary Table 2 for characteristics of the matched sample).

#### Emotional Face-matching Task

During functional MRI (fMRI) scanning, participants completed an emotional face-matching task. The face-matching task is an implicit emotion processing task which has previously been shown to robustly engage the amygdala.^50, 51^ This task includes two conditions: an emotional condition (matching faces) and a control condition (matching shapes). In the emotional condition, subjects view trios of faces with fearful or angry expressions and are asked to match the two corresponding stimuli illustrating the same individual. In the control condition, the participants view trios of simple geometric shapes (circles, vertical and horizontal ellipses) and are asked to match the two corresponding geometric shapes. The task is presented in eight blocks of six trials (30 s) with alternating epochs of face- and shape-matching conditions (task duration: 4.3 min or 130 whole-brain scans).

#### Imaging parameters

Blood oxygenation level-dependent fMRI was performed using three identical scanners (Siemens Trio 3 T; Siemens Medical Solutions, Erlangen, Germany) at the Central Institute of Mental Health Mannheim, University of Bonn and the Universitätsmedizin Charité, Berlin. Data were acquired with gradient-recalled echo-planar imaging (GRE-EPI) sequences with the following parameters: TR 2000 ms, TE 30 ms, 28 oblique slices (descending acquisition) per volume, 4 mm slice thickness, 1 mm slice distance, 80° flip angle, 192 mm FOV, and 64 × 64 matrix. Quality assurance measures were conducted on every measurement day at all sites according to a multicenter quality assurance protocol revealing stable signals over time.

#### Functional Imaging Processing

fMRI images were processed using Statistical Parametric Mapping (SPM8, http://www.fil.ion.ucl.ac.uk/spm/). The procedures followed our previously published studies with the same task.^52, 53^ In brief, the preprocessing included realignment, slice timing correction, normalization to the Montreal Neurological Institute (MNI) space with voxel size 3 × 3 × 3 mm^3^, and spatial smoothing with a 9 mm full-width at half-maximum (FWHM) Gaussian kernel. The preprocessed images were then analyzed at two levels. At the first level, images for each individual were analyzed using general linear models (GLM), where the boxcar vectors for task conditions (convolved with the standard SPM hemodynamic response function) were included as regressors of interest and the six head motion parameters from the realignment step were included as regressors of no interest. The data were high-pass filtered (cutoff, 128 s) and individual maps for the ‘face-matching>shape-matching’ contrast were computed. The contrast images were then used for a second-level random effects analysis. To test for genetic association, these contrast images were analyzed using the multiple regression model including the three allelic groups (labelled as 0,1,2) as variable of interest and age, sex and scanner site as the nuisance covariates. Significance was measured at *P*<0.05 family-wise error corrected across an *a priori* defined anatomical mask of the bilateral amygdala from the Automated Anatomical Labeling atlas.^54^ To probe more precisely which subregion the peak voxel was located, we further extracted three amygdala subdivisions (superficial, latero-basal and centro-medial complex) from the Anatomy toolbox^55, 56^ and corrected the peak voxel across the three subregional masks. The corrected *P*-values for each of the masks were reported.

### Healthy subjects for expression quantitative trait loci analysis

To identify the impact of risk SNPs on mRNA expression, we utilized a well-characterized gene expression database BrainCloud (http://braincloud.jhmi.edu/).^57^ The data in BrainCloud is aimed at increasing our understanding of the regulation of gene expression in the human brain and will be of value to others pursuing functional follow-up of disease-associated variants. The BrainCloud is comprised of 261 postmortem dorsolateral prefrontal cortex of non-psychiatric normal individuals, including 113 Caucasian subjects and 148 African American individuals across the lifespan. We used 224 postnatal individuals (110 Caucasians and 114 African Americans) from BrainCloud which contains the genotype data. The raw genotype data were obtained from BrainCloud; expression data and demographic information such as RNA integrity number, race, sex, and age were also obtained. The prenatal subjects were removed from the expression quantitative trait loci (eQTL) analysis since *PCDH17* mRNA expression is differentially expressed in fetal subjects compared with postnatal subjects. The statistical analysis was conducted using linear regression, with RNA integrity number, sex, race and age as covariates.

### RNA-seq data processing in SMRI data set for diagnostic analysis

We downloaded raw RNA-sequencing reads from the SMRI data set (http://sncid.stanleyresearch.org/) in the FASTQ file format. The RNA-seq data were from frontal cortex (15 BPD, 15 MDD and 15 healthy controls) generated by SMRI neuropathology collection. Reads after adaptors and low quality filtering using btrim64^58^ were aligned to human reference genome (hg38, http://asia.ensembl.org/index.html) through Tophat2 v2.0.14 (ref. 59) with mismatches, gap length as well as edit distance all no more than 3 bases. Cufflinks v2.2.1 (ref. 60) was then applied to call new transcripts and quantify both the new and old ones with default parameters. For replicate samples, accepted hits bam files from Tophat2 alignment were merged by Samtools v0.1.18 (ref. 61) and the merged files were utilized for the following Cufflinks quantification. Only reads uniquely mapped to genes were used to calculate the gene expression level. To quantify mRNA expression, FPKM (Fragments per Kilobase per Million mapped reads) was calculated to measure gene-level expression according to the formula: FPKM=*R* × 10^3^/*L* × 10^6^/*N*; where *F* is the number of fragments mapping to the gene annotation, *L* is the length of the gene structure in nucleotides, and *N* is the total number of sequence reads mapped to the genome of chromosome.

Statistical analyses of mRNA expression associated with diagnosis were conducted in R 3.0.1 using linear regression, covaring for RNA integrity number, sex, age, race, duration of illness, brain pH, post-mortem interval, suicide status and batch number in each sample. All reported two-sided *P*-value s were calculated from *t* statistics computed from the log fold change and its standard error from each multiple regression model, and therefore represent covariate-adjusted *P*-values.

### Pluripotent stem cell analysis

The expression analysis of *PCDH17* in iPSCs and neurons derived from BPD patients and healthy controls has been described in a previous study.^62^ In brief, subjects contributing a skin sample were from a psychiatric clinic in a mid-western college city, Caucasian, and were diagnosed with Bipolar I Disorder, or healthy unaffected controls were ascertained through advertising on the University of Michigan Clinical Studies website. To characterize the iPSC and to determine whether there were differences in their gene expression profiles with neuronal differentiation, total RNA was isolated from six individual iPSC cell lines (3 BPD patients and 3 controls) before and following 8 weeks of neuronal differentiation using the TRIzol reagent (Invitrogen, Grand Island, NY, USA). The RNAs were amplified and hybridized to GeneChip U133 Plus 2.0 microarrays (Affymetrix, Santa Clara, CA, USA). Only complete sets of iPSC (three BPD patients and three controls) and neurons (same three BPD patients and same three control cell lines from six individuals) were analyzed to minimize stochastic changes due to culture conditions. Detailed protocols about fibroblast derivation, iPSC derivation and neuronal differentiation were described previously.^62^

### Plasmid constructs and reagents

The pCMV (Agilent Technologies, Santa Clara, CA, USA) encoding human *PCDH17* with a C terminus Myc-tag and pEGFP vector were used. The integrity of constructs was verified by sequencing. The following antibodies were used: GFP rabbit polyclonal (MBL) and Myc mouse monoclonal (MBL).

### Cortical neuronal cultures and transfection

Dissociated cortical neurons were prepared from cerebral cortex of C57BL/6J mice embryonic (E16.5). In brief, cortices were dissected, trypsinized and gently minced. Neurons were seeded to a density of 1 × 10^6^ viable cells/35 mm glass bottom dishes previously coated with poly-D-lysine (1 mg ml^−l^) for at least 12 h at 37 °C. Cultures were maintained at 37 °C with 5% CO_2_, supplemented with Neurobasal medium with 2% B27 (Invitrogen), penicillin/streptomycin (100 U ml^−1^ and 100 μg ml^−l^, respectively), 2.5 mM glutamine, and 5% fetal bovine serum. Cultures were transfected with Lipofectamine 2000 (Invitrogen) at 17–18 days *in vitro* (DIV) with EGFP plus mock plasmid or PCDH17-myc and maintained for additional 1 day before imaging analysis.

### Quantitative morphological analysis of dendritic spines

Transfected neurons were fixed in 4% paraformaldehyde with 4% sucrose at 4 °C. Immunostaining with antibody to GFP was used to circumvent potential unevenness of GFP diffusion in spines. For co-transfection experiments, the neurons that clearly transfected with both GFP and PCDH17-myc were captured as images. Transfected neurons were chosen randomly and images were obtained using a TCS SP8 confocal microscope (Leica Microsystems). The acquisition parameters were kept constant for all scans in the same experiment. Deconvolution was performed and image stacks (0.13 mm z series) were quick projected. The first or second dendrites that were arborized from a neuron were subjected to morphological analysis. Data analysis was carried out using ImageJ software (NIH, MD, USA). Dendritic spine density was evaluated manually. Individual spines on dendrites were traced and neck length and head width of each spine was measured. To analyze spine morphology, at least 400 spines (from 16 neurons) were measured for each condition. On the basis of morphology, spines were classified into the following categories: (1) Thin, where the head width was <0.4 μm; (2) Mushroom, where the head width was >0.4 μm; and (3) Stubby, where the neck length was <0.1 μm. Statistics were calculated in Prism v 6.07 (GraphPad Software, San Diego, CA, USA). Spine density, neck length and spine width between two groups were compared using two-sided student’s *t*-test. To compare the proportion of different spine types between two groups, two-way analysis of variance with Bonferroni *post hoc* test were used.

## Results

### Overview of research strategy and experimental design

**Figure 1:**
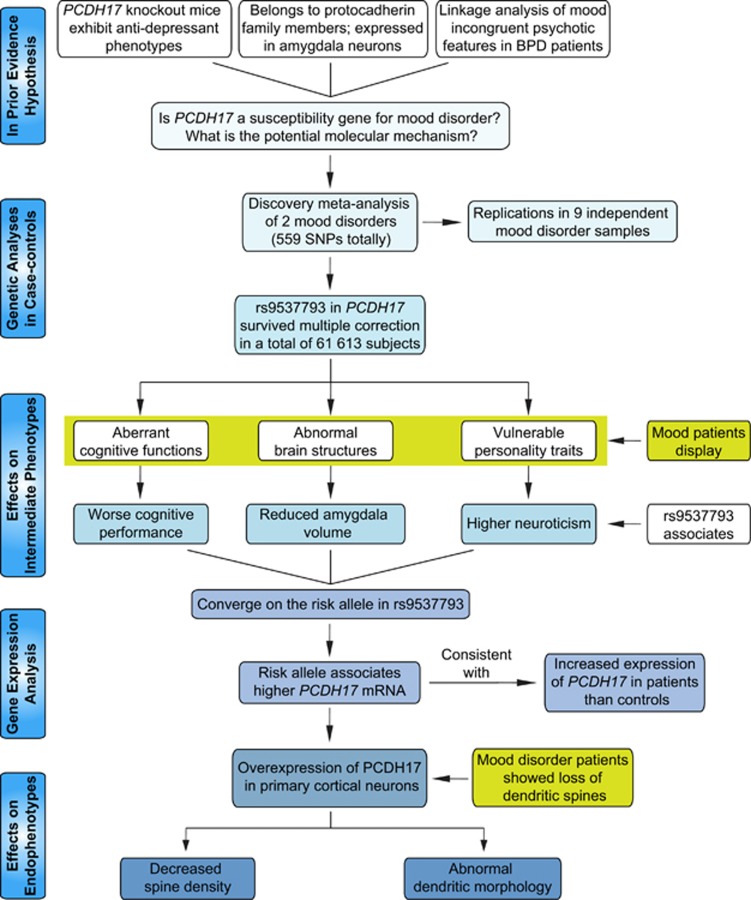
Overview of convergent research strategy and experimental design. The strategy began with clinical genetic association, replication in independent data sets and then meta-analysis of the pooled data. Positive single-nucleotide polymorphism (SNPs) were then tested on biological intermediate phenotypes in human populations and on gene expression in human brain in patients and healthy controls. The latter analyses identified a higher expression of *PCDH17* as a risk factor for major mood disorders, which was then characterized the function in cell culture. BPD, bipolar disorder.

The sequence of hypothesis-driven experiments and tests leading to converging evidence, from initial genetic screening to genetic association with aspects of human cognition, personality, brain structure and function and gene expression to confirmatory experiments in tissue culture, is summarized in Figure 1.

### Identification of rs9537793 on 13q21.1 associated with major mood disorders

**Figure 2:**
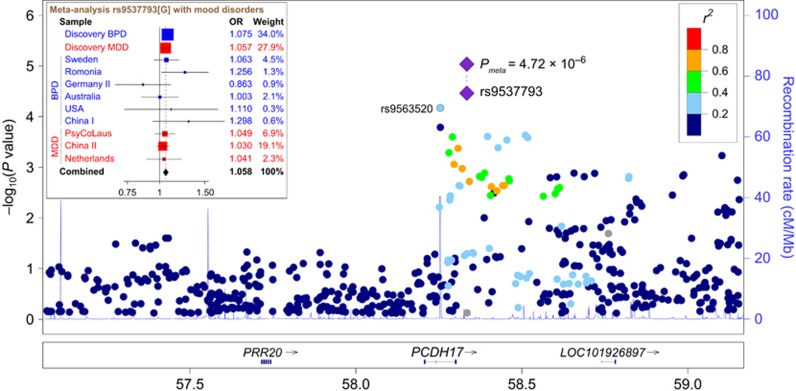
Genetic association of *PCDH17* with risk for major mood disorders. A physical map of the region is given and depicts known genes within the region. BPD, bipolar disorder; MDD, major depressive disorder.

With the use of a BPD GWAS^27^ (10 410 cases and 10 700 controls) and a MDD GWAS^28^ (9227 cases and 7383 controls) in European populations (with no overlap in control subjects between the two samples as stated in the original GWASs), we performed a meta-analysis of 559 SNPs covering 2.0 Mb in the 13q21.1 region to test whether any markers show region-wide significant associations. One SNP, rs9537793, in the 3’ downstream of *PCDH17* showed the highest association with mood disorders among all the 559 variants (*P*-value=3.30 × 10^–5^, OR=1.067, Figure 2), followed by rs9563520 (*P*-value =8.72 × 10^–5^, OR=1.086). These two SNPs survived Bonferroni correction according to the number of analyzed SNPs (*n*=559), and are in low linkage disequilibrium (LD) in European populations (*r*^*2*^=0.21). Furthermore, none of the other tested SNPs on 13q21.1 was in high LD with rs9537793 (all *r*^*2*^<0.8), which was also supported by a higher density LD analysis in 1000-Human-Genome European samples (Supplementary Figure 1).

To further confirm the observed associations in the discovery meta-analysis, we selected the top two SNPs (rs9537793 and rs9563520) and conducted replication analyses in a large collection of nine independent BPD and MDD case–control samples. The final replication sample sets included 9920 patients and 13 973 controls (Supplementary Table 1). Although the sample sizes and ascertainment strategies differed from those of the discovery meta-analysis, we also observed a significant association of mood disorders with rs9537793 in these replication samples (*P*_rep_=1.70 × 10^–2^, OR=1.043). To increase the power of statistical association, we conducted a meta-analysis on the discovery and replication mood disorder samples using the R package (metafor). This meta-analysis showed that rs9537793 was significantly associated with major mood disorders in a total of 29 557 cases and 32 056 controls (*P*_meta_=4.72 × 10^–6^, OR=1.058, Figure 2), with no heterogeneity among individual samples (*P*_heterogeneity_=0.560, *I*^*2*^=0). A separate analysis found that rs9537793 was significantly associated with BPD (*P*_BPD_=1.40 × 10^–4^, OR=1.073) and MDD (*P*_MDD_=6.08 × 10^–3^, OR=1.046), respectively. The results for each replication sample were shown in Supplementary Table 3. Taken together, the association analyses suggest that rs9537793 on 13q21.1 may confer genetic risk towards major mood disorders. However, rs9563520 was not significant in the replication samples (Supplementary Table 3), and was therefore dropped from further analyses.

### Effect of rs9537793 on cognition, personality, amygdala structure and function

It has been reported that *PCDH17* is expressed in amygdala neurons,^24^ and we previously have shown that the gene is enriched along corticobasal ganglia synapses in a zone-specific manner during synaptogenesis.^25^ Here, with the use of GTEx (Genotype-Tissue Expression project),^63^ a RNA-seq resource comprising diverse human tissue samples, we showed that *PCDH17* is abundantly expressed in brain areas (Supplementary Figure 2), such as amygdala, caudate, prefrontal cortex and hippocampus *etc.* These brain regions subserve memory and emotional processes, and have been frequently implicated in the neuropathology of major mood disorders.^3^ We therefore hypothesized that if the risk-associated SNPs affected the biology of these brain regions, then deficits in cognition or other functions mediated by these areas would also be associated with the risk genotypes.

We first tested the effects of the risk SNP rs9537793 on educational attainment, a ‘proxy phenotype’ of general cognitive abilities (correlation r~0.5).^34^ The first studied sample comprised 95 427 individuals for ‘College’ and 101 069 for “EduYears”. Notably, rs9537793 was significantly associated with “EduYears” (Beta=–0.011, *P-*value=6.35 × 10^–3^, Supplementary Table 4), and ‘College’ (OR=0.970, *P*-value =1.04 × 10^–3^), with the risk allele indicating lower educational levels.^34^ Furthermore, in another overlapping sample with more subjects (*n*=293 723) but only focusing on the ‘EduYears’ phenotype,^36^ the association for rs9537793 was substantially strengthened (Beta=–0.016, *P-*value=2.68 × 10^–10^).

Considering the abundant expression of *PCDH17* in the amygdala and caudate, recognized as key regions for emotional processing,^64, 65, 66^ we then examined whether the risk SNP was also associated with personality traits related to emotion. In the first sample including 63 661 subjects,^40^ we found rs9537793 associated with neuroticism (Beta=0.014, *P-*value=1.59 × 10^–2^, Supplementary Table 5), an important emotional trait that was significantly associated with mood disorders.^38^ Later, in an overlapping cohort with larger sample size (*n*=170 911),^41^ the association between rs9537793 and neuroticism was strengthened (Beta=0.010, *P-*value=4.59 × 10^–3^). In these series of analyses, the rs9537793 risk allele carriers tend to show increased vulnerable personality traits compared to protective allele carries.

We also analyzed whether the risk SNP would relate to changes in brain structure. Using region of interest analyses in MRI-based morphometry of 13 171 individuals,^46^ we observed significant volume decreases for risk allele carriers of rs9537793 in amygdala (Beta=–5.92, *P-*value=9.95 × 10^–3^, Supplementary Table 6) and hippocampus (Beta=–9.08, *P-*value=4.74 × 10^–2^), brain regions responsible for emotional reactions and memory processing which often show volumetric reductions in patients with mood disorders.^67^

**Figure 3:**
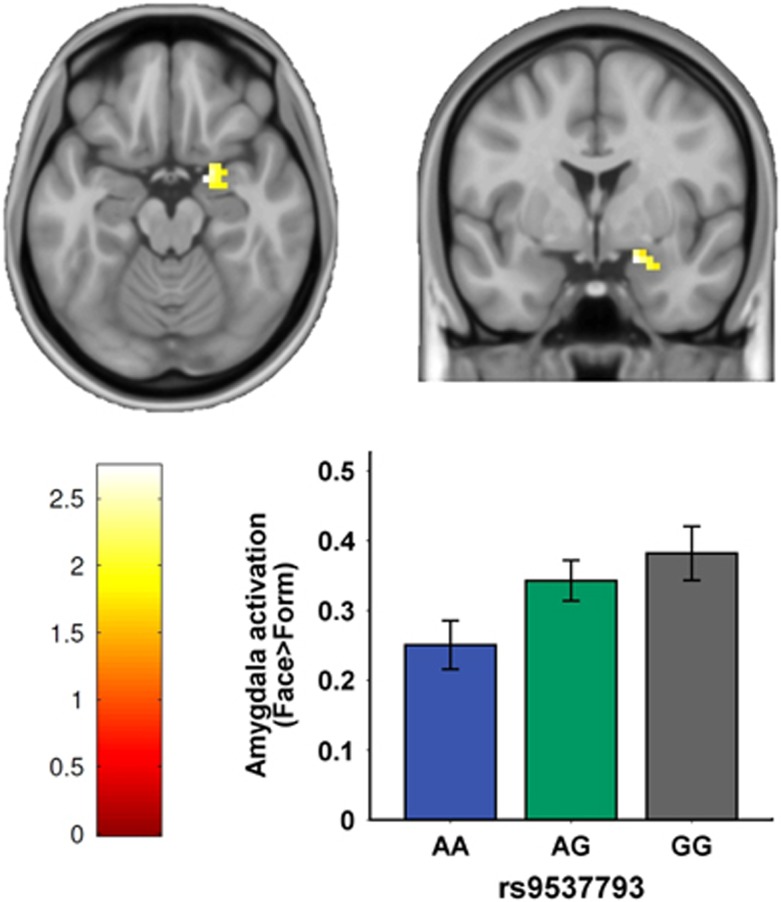
Effect of the risk SNP rs9537793 on amygdala function. During emotional face-matching task, carriers of the risk allele (G) of rs9537793 exhibited significantly increased allele-dosage-dependent activation in the right amygdala (*T*=2.74, small-volume family-wise error (few) corrected *P*-value =0.046). Number of subjects in each group: AA=77, GA=146, GG=74. SNP, single-nucleotide polymorphism.

Significant associations of rs9537793 with emotional traits and amygdala volume lead us to hypothesize this SNP may also be associated with amygdala function underlying negative emotional processing. Thus, we employed fMRI to test the effects of risk-associated SNPs on amygdala activity during negative emotional task, given the prior evidence that patients with mood disorders have previously been shown to exhibit elevated amygdala activity in response to negative emotional stimuli.^68^ There also is evidence that individuals at increased genetic risk for bipolar disorder show similar patterns of amygdala activity, suggesting that amygdala hyperactivity may reflect a neural mechanism of genetic risk for mood disorders.^69^ We hypothesized that healthy subjects who are carriers of the risk-associated allele of *PCDH17* would have increased amygdala activity in response to negative emotional stimuli (in an emotional face-matching task) compared with carriers of the protective allele. With focus on amygdala activity, we conducted a region of interest analysis on 297 subjects who underwent this negative emotional face task during fMRI scanning. This analysis revealed a significant linear effect of rs9537793 on the activity of the right amygdala for the task contrast (that is, emotional face-matching vs shape-matching; *T*=2.74, small-volume family-wise error corrected *P*-value =0.046, Figure 3). Homozygous risk allele carriers of rs9537793 (that is, GG) showed the highest amygdala activity, followed by heterozygotes and protective allele (that is, A allele) homozygotes (Figure 3). This association is also consistent with previous evidence of higher amygdala activity in patients with mood disorders than healthy controls in response to negative emotional stimuli.^68^ However, rs9537793 did not show evidence of association with the left amygdala activity. The laterality of this amygdala difference is in agreement with the proposed general role of right hemisphere brain regions in processing faces, as well as with recent reports implicating a specific role for the right amygdala in processing angry and fearful facial expressions.^50, 70^ Additional analyses indicated that the effect of rs9537793 on right amygdala activity was independent of demographic characteristics (for example, age, sex etc), and the genotype groups did not differ in performance (accuracy and reaction time) on the emotional task, indicating that general attentional, perceptual, and cognitive phenomena did not contribute to the observed amygdala differences.

Collectively, these data suggest that the *PCDH17* rs9537793 not only associates with clinical diagnosis of major mood disorders, but also affects the specific intermediate phenotypes. These results shed light on the potential neuronal mechanism of *PCDH17* in the illnesses and neurodevelopment.

### Risk genotypes in rs9537793 and diagnosis predict PCDH17 mRNA expression

**Figure 4:**
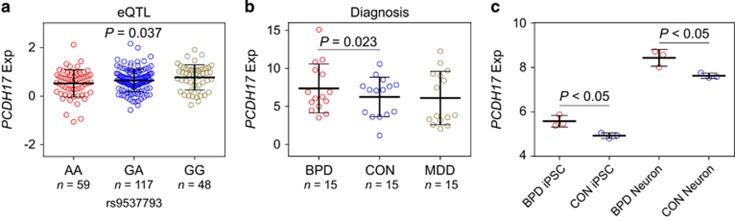
Risk genotype and diagnosis predict *PCDH17* expression. (**a**) Association of rs9537793 with *PCDH17* expression in the 224 postnatal subjects from frontal cortex in BrainCloud data set. (**b**) Diagnostic analysis of *PCDH17* expression in adult samples from frontal cortex. (**c**) Expression analysis of *PCDH17* in iPSCs and neurons derived from BPD patients and healthy controls (each *n*=3). BPD, bipolar disorder; CON, healthy controls; eQTL, expression quantitative trait loci; MDD, major depressive disorder.

The associations of rs9537793 with major mood disorders and with related intermediate phenotypes in multiple independent samples lend statistical and neurological support to the involvement of this genomic locus in the risk of illness. However, these findings do not identify the underlying molecular mechanism. Rs9537793 is in low LD with its surrounding SNPs (Supplementary Figure 1), and within 400 kb around rs9537793 there is only *PCDH17* gene, hence we focused on this gene in the following analyses. In the existing human mRNA databases (Ensembl and UCSC), there is only one known *PCDH17* protein-coding transcript, which is also further confirmed by junction-level analysis in brain RNA-sequencing data from ENCODE and GTEx, which has been described in our previous study.^71^ In brief, junctions are the RNA-sequencing read counts that span at least two exons, and junction reads between nonadjacent exons (exon-skipping junctions) are indicators of alternative splicing. In *PCDH17* such junctions are quite rare. We thus investigated this *PCDH17* canonical transcript expression in brain tissues and its relationship to our evidence of genetic risk. We utilized BrainCloud,^57^ a well-characterized gene expression database which contains genetic variation and mRNA data in human frontal cortex. Interestingly, the risk SNP rs9537793 was associated with *PCDH17* mRNA expression in 224 healthy postnatal individuals (*P-*value=0.037, Figure 4a), with the risk allele indicated higher mRNA levels.

The eQTL analyses showed a potential molecular mechanism for genetic risk, but not association with illness *per se*. To gain further insight into the potential pathophysiological roles of *PCDH17*, we assessed the effects of diagnostic status on the expression changes of *PCDH17* mRNA. We analyzed the RNA-seq data of frontal cortex in 15 BPD patients, 15 MDD cases and 15 healthy controls. Although the *PCDH17* is not increased in patients with mood disorders (BPD and MDD combined) compared to healthy controls (data not shown), it should be noted that gene expression could easily be downstream changes from a variable genetic (or environmental) influence, we therefore separated BPD and MDD samples and compared *PCDH17* expression in each diagnostic group with healthy controls, respectively. Interestingly, we found expression of *PCDH17* to be significantly higher in individuals with BPD compared to healthy controls (*P-*value=0.023, Figure 4b), which is consistent with the eQTL analysis of higher mRNA levels in individuals carrying the risk alleles. In addition, in a previous thalamic transcriptome study^72^ using the samples from SMRI neuropathology collection (*n*=15 each, schizophrenia/BPD/MDD/controls), *PCDH17* expression was again increased in patients with BPD versus healthy controls (*P-*value=0.047, Supplementary Figure 3). However, *PCDH17* expression did not differ between MDD patients and healthy controls in these samples.

The involvement of *PCDH17* in the development of BPD was also supported by the expression analysis in BPD patients-derived induced pluripotent stem cells (iPSCs) and neurons. It has become apparent that iPSCs provide the opportunity to study the stepwise differentiation of patient derived cells into neurons to identify alterations in cell behavior and test novel therapeutic approaches.^73, 74^ Mood disorders are increasingly recognized as neurodevelopmental disorders, and subtle alterations in gene expression and pathways in early developmental events can produce neurological consequences that only become apparent much later in life.^75^ We therefore examined changes in *PCDH17* expression during iPSCs derived from well-characterized patients differentiate into neurons (BPD *n*=3; Control *n*=3) using data from a previous study.^62^ Interestingly, *PCDH17* expression tends to show upregulation during this stage of neuronal differentiation in both BPD patients (BPD iPSCs versus BPD neurons, *P*-value <0.001) and control cell systems (control iPSCs versus control neurons, *P*-value <0.001) (Figure 4c), suggesting this gene may be involved in early brain development. Further, diagnostic analysis found that expression of *PCDH17* was higher in BPD patients than healthy controls in both iPSCs (BPD iPSCs versus control iPSCs, *P*-value <0.05) and neurons (BPD neurons versus control neurons, *P*-value <0.05, Figure 4c).

Taken together, our data support the hypothesis that the mechanism by which the disease-associated SNPs contribute to risk for BPD and related phenotypes involves the regulation of *PCDH17* transcription and further suggest that overexpression of this gene would result in the biological effects related to the illness pathogenesis. The consistent case–control differences observed for *PCDH17* in BPD suggest that the molecular biology of this gene involves broader elements of BPD pathogenesis than just that of genetic risk. In contrast, the *PCDH17* expression is not altered in MDD patients, implying the link between *PCDH17* and MDD is primarily on its genetic risk level, and further studies are necessary to validate this result.

### Elevated PCDH17 expression results in decreased spine density and abnormal spine morphology

Altered synaptic connectivity and plasticity have been repeatedly reported in patients with mood disorders.^76^ Changes in dendritic spine number and morphology are tightly coordinated with synaptic function and plasticity,^77, 78^ and loss of dendritic spines in the brain has been seen in both individuals with BPD and individuals with MDD,^4^ making it served as a common potential substrate for the illnesses.

**Figure 5:**
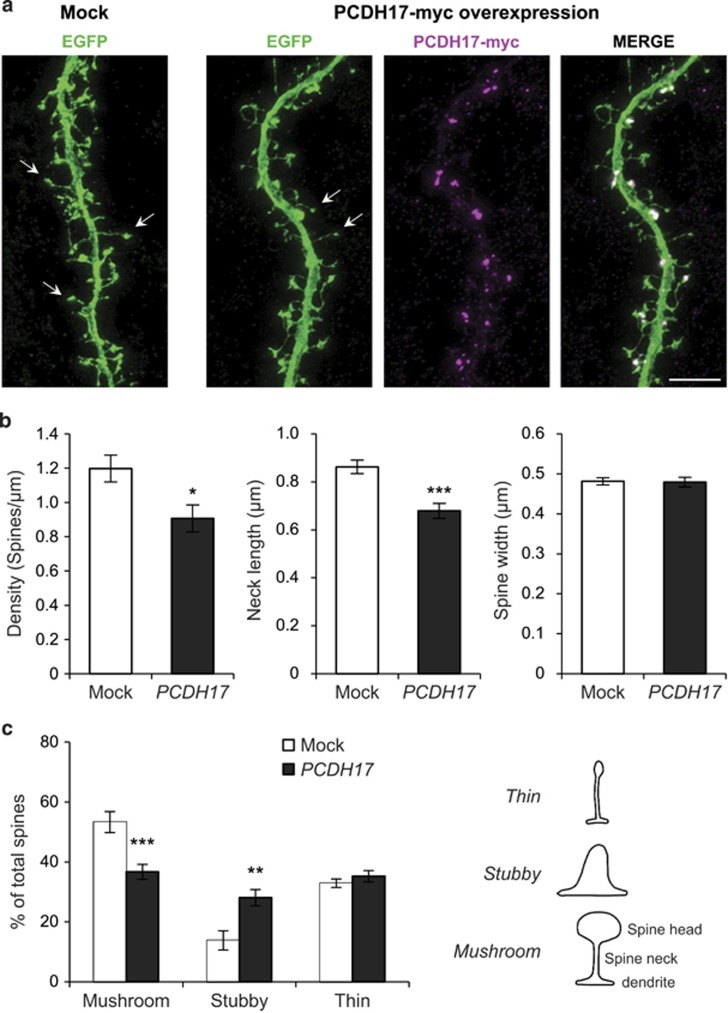
Overexpression of *PCDH17* decreases spine density and results in abnormal spine morphology in cultured cortical neurons. Scale bars represent 5 μm. (**a**) Cultured cortical neurons were transfected at DIV17-18 with EGFP plus mock plasmid or *PCDH17*-myc and maintained for additional one day. Neuronal morphologies were visualized by EGFP. Representative spines were arrowed in both groups. (**b**) Quantification of dendritic spine parameters (density, neck length and spine width). (**c**) Dendritic spines were divided in three different categories depending on their morphology: stubby, thin and mushroom, as indicated in the line drawing on the right. The diagram showed the percentage of total spines belonging to each category in Mock or *PCDH17*-Myc transfected cortical neurons. Dendritic spines were counted for each condition from four separate cultures. Error bars indicated s.e.m. *P<0.05, **P<0.005, ***P<0.001; Student's two-sided *t*-test (**b**) and two-way analysis of variance *post hoc* Bonferroni test (**c**).

It is thus reasonable to hypothesize that *PCDH17* would affect the density or morphology of dendritic spines, where excitatory synapses are placed.^79^ Accordingly, we investigated the onset of a possible effect of *PCDH17* on dendritic spine density and morphology *in vitro* by confocal imaging analysis (Figure 5a). As expected, overexpression of *PCDH17* significantly decreased spine density compared with the mock group (mock, 1.198±0.078 spines per μm; overexpression of *PCDH17*, 0.907±0.079 spines per μm; *P*-value <0.05; Figure 5b). Intriguingly, increased expression of *PCDH17* also dramatically affected the morphology of dendritic spines, that is, reduced the spines neck length compared with mock group (mock, 0.862±0.028 μm; overexpression of *PCDH17*, 0.679±0.031 μm; *P*-value <0.001; Figure 5b). However, the spine width did not differ between mock group and the *PCDH17* overexpression group (mock, 0.481±0.009 μm; overexpression of *PCDH17*, 0.480±0.012 μm; *P*-value >0.5; Figure 5b).

For a more detailed morphological analysis, dendritic spines were categorized according to their shape (mushroom, stubby and thin) using a highly validated classification method (see Materials and methods section). As shown in Figure 5c, overexpression of *PCDH17* induced a significant reduction in the proportion of mushroom spines (*P*-value <0.001) and a concomitant significant increase in the proportion of stubby spines (*P*-value <0.005), while the proportion of thin spines did not differ between groups (*P*-value >0.5).

The above data suggests that elevated *PCDH17* affects the structure and density of dendritic spines, which will result in the changes of synaptic transmission and plasticity. These results further suggest a potential neuronal mechanism for the neuropathology of *PCDH17* in mood disorders as well as dysfunctional memory, emotion and brain functions observed in patients.

## Discussion

We report a genetic association with mood disorders within the genomic region spanning *PCDH17*. To move beyond statistical association with clinical diagnosis and to obtain convergent evidence for association between *PCDH17* and mood disorder related biology, we have performed a series of convergent experiments testing the effects of risk-associated SNPs on several intermediate biological phenotypes. A consistent pattern of allelic association, involving cognition, neuroticism as personality trait, structural and functional imaging, was found in independent samples and in expression of the *PCDH17* gene in brain tissues. The likelihood that the same risk-associated allele would predict by chance variation in each of investigated phenotypes across diverse samples and always in the direction of abnormality is remote. To our knowledge, to date, the region of interest on chromosome 13q21.1 has not been reported as a major locus in the few GWAS of mood disorders.^10, 12, 13, 14, 15^ Those negative results may reflect the fact that the sample size of individual GWAS is still small, as our meta-analysis combining the GWAS and independent replication samples was necessary to detect associations of *PCDH17* SNPs. Conversely, the *P*-value s reported here would not be significant if we corrected for all SNPs in the genome, and further validations in larger samples are necessary. *PCDH17* belongs to the protocadherin gene family, a subfamily of the cadherin superfamily.^80^ The protein encoded by *PCDH17* may have a role in the establishment and function of specific cell−cell connections in the brain. Notably, the identification of *PCDH17* as a susceptibility gene for major mood disorders is also consistent with a prior report of another cadherin gene (*FAT*) as a potential risk locus for BPD.^81^

Our results also provide support for the previous epidemiologic evidence of genetic overlap between BPD and MDD. However, our data do not explain why, among all those carrying risk alleles, some develop BPD, others develop MDD and still others remain apparently healthy. This phenomenon may reflect variation at a larger number of genetic loci, only few of which have been detected to date, environmental influences, and perhaps epigenetic factors. The genetic architecture of major mood disorders seems to be polygenic and/or highly heterogeneous, and the genetic association findings so far seem to account for little of the inherited risk for mood disorders. As robust findings accumulate and sample sizes grow, the identified genes may highlight pathways of etiologic relevance. In addition to major mood disorders, *PCDH17* is also implicated in a previous schizophrenia study,^82^ in which *PCDH17* was significantly increased in the brain of schizophrenia patients. This is intriguing, as is usually the case, genes are not specific for a psychiatric diagnosis, but are more like a combinatorial building-block (Lego-like) structure underlies psychiatric syndromes,^83^ and further studies are necessary to test the contributions to *PCDH17* to other neuropsychiatric disorders.

Exploring the potential effects of elevated *PCDH17* levels on the morphology and density of dendritic spines has noteworthy implications for both normal brain function and the pathophysiology of mood disorders. Synaptic dendritic spines are dynamic structures that regulate neuronal responsiveness and plasticity, and changes in dendritic spine number and morphology accompany synapse formation, maintenance and elimination, allowing the establishment and remodeling of connectivity within neuronal circuits.^84^ Elimination of dendritic spines has been observed in patients with BPD and MDD, as well as in cellular or animal models by overexpressing/knockout risk genes for mood disorders such as *NRG3*, *DISC1*, *NRG1* and others.^85, 86, 87, 88^ In this study, we reported elevation in *PCDH17* expression results in the loss of dendritic spines, which might be able to explain the observed association of *PCDH17* with multiple brain functions and to implicate a potential molecular and neuronal mechanism of *PCDH17* involvement in the risk for mood disorders. Since the precise mechanisms detailing how *PCDH17* affects the dendritic spines and related synaptic functions remain unknown, further work is therefore needed.

The discovery of *PCDH17* in the risk of mood disorders may also have therapeutic implications. Previous reports showed that dendritic spines may serve as a common substrate for many psychiatric disorders, particularly those that involve cognitive deficits such as BPD and MDD. However, as spine modifications are associated with cognitive function, spine deficits may be more relevant for some cognitive symptoms or intermediate phenotypes than others. Given the heterogeneity of these complex disorders, some individuals may exhibit more marked spine phenotypes, particularly those with more severe cognitive deficits. Further studies of human neuropathology should strive to understand the degree of correlation between severity of cognitive deficits and dendritic spine dysmorphogenesis. The risk genes that control spines provide a future direction for understanding how these genes disrupt synaptic function, neuronal circuit organization and behavioral output in a disease-specific manner. Thus, investigating the mechanisms can uncover future candidate genes and identify the best molecular candidates for therapeutic targeting. Indeed, many of the proteins in synapses are enzymes that could be manipulated with designer small molecules and drugs that target trophic and morphogenic signaling pathways may prove to be more effective, as they could alter cellular connectivity and induce fewer side effects. New drugs may be designed to prevent the emergence of symptoms in genetically susceptible individuals, delay the progression of symptoms in the early stages of the disease, or mitigate symptoms or promote functional recovery after the disease is fully manifested. Specifically, drugs that target dendritic spine regulation might aim to promote spine maturation and restore spine stability, to fortify existing synapses and restore spine plasticity, or to prevent synapse loss in mood disorders.

However, there are several limitations in the present study, and we are cautious in the interpretation of the results. First, we noticed that the association *P*-value s between rs9537793 and major mood disorders did not achieve the conventional genome-wide level of statistical significance (*P*=5.00 × 10^–8^), and give the herein observed OR (1.058), the results would not become genome-wide significant until the sample size increases to 102 786 cases and 102 786 controls (have a power of >80%). However, as mentioned in the previous aggregated analyses,^15, 16^ BPD and MDD are polygenic disorders involving hundreds of thousands alleles in it with each allele showing minor effect, and there might be true findings among those markers only passing nominal significance in the initial GWAS, but later were confirmed in independent samples. Of note, we did not set up any *in prior* hypothesis of choosing or dropping any SNPs 13q21.1, but selected all the analyzed SNPs (*n*=559) from previous GWAS data sets^27, 28^ in this genomic region without any bias. Although the result did not achieve genome-wide significance, it does survive multiple correction according to the number of tested SNPs (*n*=559) in this study (corrected *P*=2.64 × 10^–3^). Second, we realized that only two brain regions (amygdala and hippocampus) were analyzed in the imaging analysis, further studies focusing on more brain regions, such as prefrontal cortex and caudate, are needed. Furthermore, we acknowledged that gene expression, eQTL data, and primary neuronal cultures were performed using tissues from prefrontal cortex, while structure and functional MRIs mainly focused on amygdala region. Further analyses of gene expression and primary neuronal cultures involving with amygdala tissues (neurons) would strengthen the present study. Third, we were aware that rs9537793 was associated with genetic risk of both BPD and MDD, but diagnostic differential expression of *PCDH17* was only observed in patients with BPD. This inconsistency is not unexpected as gene expression could easily be influenced by genetic, epigenetic and environmental factors. It is thus possible that while *PCDH17* confers risk for both BPD and MDD in the genetic level, its expression in those patients are also affected by varied factors associated with the specific pathogenic processes of these two disorders, resulting in inconsistent results between genetic analyses and gene expression profiles. However, further studies are necessary to validate this contention.

In conclusion, we have identified and characterized a novel gene with a potential genetic association with risk for mood disorders. Individual carriers of mood disorders risk alleles show shifts in cognitive performance, emotional stability (neuroticism), amygdala structure and functions during negative emotion processing in the same direction as mood disorder patients. The mechanism of these risk associations seems to be related to the genetic regulation of *PCDH17* expression, which has critical functions on the morphology and structure of dendritic spines. Together, these results may provide new insights into the etiology of mood disorders and a fresh direction for therapeutic development.
