# Supplementary material for: The protocadherin 17 gene affects cognition, personality, amygdala structure and function, synapse development and risk of major mood disorders
Source: Mol Psychiatry. 2017 Jan 10;23(2):400–12. doi: 10.1038/mp.2016.231 (PMC5794872; doi:10.1038/mp.2016.231)
Supplement: Supplementary Material [file mp2016231x1.pdf]

Replication Mood Disorder Sample Information

Among all the tested samples, both mood disorder patients and healthy controls provided written informed consent prior to their inclusion in the respective studies. All protocols used in the original studies reporting these samples were approved by the relevant ethical review bodies, and followed all applicable institutional, national and international guidelines.

Sweden BPD sample

This sample consisted of 1,415 patients with BPD (62.5% female, age ± SD = 53 ± 14, BPD type I =578, BPD type II= 517, NOS=281, SAB = 39, unknown subtype = 4), and 1,271 healthy controls (50.3% female, age ± SD= 59 ± 11 years). All subjects were unrelated to each other and ethnically Swedish. Patients with BPD were collected from the Swedish National Quality Assurance Registry for bipolar disorder (BipoläR), to which all patients with a DSM-IV diagnosis of bipolar I, II, NOS, or schizoaffective disorder are considered for registration at the participating clinics.[1] There were no other inclusion or exclusion criteria. Diagnoses were made by the treating physician with longitudinal access to all available clinical information. Controls were also identified from national population registers, and had never received a discharge diagnosis of SCZ or bipolar disorder. Controls were contacted directly in a similar procedure as the cases, gave written informed consent, were interviewed about other medical conditions and visited their family doctor or local hospital laboratory for blood donation. Patients and controls were genotyped on the Illumina Omni Express array, and the genomic inflation factor (lambda) is 1.03.

Romania BPD sample

The Romania sample consisted of 461 BPD patients and 329 healthy controls. All patients were recruited from consecutive hospital admissions and directly interviewed with the Structured Clinical Interview for DSM-IV-TR-Axis I Disorders - Patient Version (SCID-I, 1994) and the Diagnostic Interview for Genetic Studies (DIGS) version 3.0 (1999). Information provided by medical records and interviews of family members was also used in a best estimate procedure of diagnosis on the basis of DSM-IV-TR criteria. The control sample was population-based, drawn from the same population as the patients, and was screened for major psychiatric disorders prior to inclusion. The ethnicity of the patients and control subjects was determined by genealogical investigation to the grandparental generation. Only the patient sample was previously reported in other collaborative studies.[2-4] The controls were genotyped on Illumina Omni-Express chips at the Life & Brain Center in Bonn, and the patients were also genotyped on Illumina chips (partly on Omni1-Quad).

Germany II BPD sample

Cases for Germany II samples were ascertained from consecutive admissions to the psychiatric inpatient units at the Central Institute for Mental Health in Mannheim, University of Heidelberg, and at the University Hospital Würzburg as well as at other collaborating psychiatric university hospitals in Germany. DSM-IV lifetime diagnoses of BPD were assigned using a consensus best-estimate procedure, based on all available information, including semi-structured

interviews (AMDP; Germany II), medical records, and the family history method. In addition, the OPCRIT system was used for the detailed polydiagnostic documentation of symptoms.[5]

Controls for Germany II were ascertained from the population-based Heinz Nixdorf Recall Study.[6] Study protocols were reviewed and approved in advance by Institutional Review Boards of the participating institutions.

All subjects provided written informed consent and were genotyped using the Illumina platform.

Australia BPD sample

The Australia sample included 330 BPD patients and 1,811 healthy controls. Subjects were ascertained through two studies: 1) a BPD pedigree sample (described in McAuley et al.[7]) and 2) a specialized Sydney Black Dog Institute BPD clinic sample (described in Mitchell et al. 2009) [8]. All subjects were interviewed by trained research staff using the DIGS or SCID, using best-estimate DSM-IV diagnoses derived from those instruments, medical records and FIGS. First, for the pedigree sample, only one BPD subject per family was included in the case sample. Pedigrees were only included in the original genetic study if there was unilineal inheritance, and at least two BPD subjects including at least one with bipolar I disorder. Subjects were ascertained through clinical presentations to the Mood Disorders Unit at the Prince of Wales Hospital in Sydney, direct referrals from Australian clinicians, and BPD consumer organizations. Second, for the clinic sample, subjects comprised consecutive subjects referred by psychiatrists or general practitioners for specialized clinical review. All patients provided written informed consent to participate in this study and the study was approved by the local ethics committee. Patients were included in the MooDS study and genotyped at the Life & Brain Center in Bonn using the Illumina platform.

Australia controls were drawn from families participating in the Brisbane Longitudinal Twin Study, an unselected community sample recruited to take part in studies of melanoma risk factors, cognition, and other phenotypes. Subjects were not screened for any phenotype relevant to BPD. The study was approved by the ethic committee and all proband gave written informed consent. All subjects were genotyped as a single project by deCODE using the Illumina platform and have been through an extensive QC process including exclusion for non-European ancestry. The sample is overwhelmingly of northern European origin, predominately from the British Isles.

USA BPD sample

The genotype data in USA BPD sample was obtained from dbGaP accession number phs000979.v1.p1 on May 25, 2016. In brief, postmortem brains of BPD patients and healthy controls were collected at the Human Brain Collection Core, NIMH with informed consent from the legal next of kin (NIMH protocol 90-M-0142), and at the Brain and Tissue Bank for Developmental Disorders of the NICHD (contracts NO1-HD-4-3368 and NO1-HD-4-3383) and through the Stanley Medical Research Institute. All BPD patients met DSM-IV criteria for a lifetime Axis I diagnosis of BPD, and controls had no history of psychological or psychiatric problems. Genotyping was performed using HumanHap650Yv3.0 and Human1M-Duov3_B.

China BPD sample

The patients who met DSM-IV criteria for BPD type 1or type 2 were recruited from the Division of Mood Disorders at Shanghai Mental Health Center, Shanghai Jiao Tong University School of Medicine between November 2006 and October 2010. Each patient was independently interviewed and diagnosed by a consensus of at least two experienced psychiatrists. Diagnoses were further confirmed with an Extensive Clinical Interview and a Structured Clinical Interview for DSM-IV Axis/Disorders, Patient Version (SCID-P) given by a research psychiatrist. Subjects with comorbid diagnosis of other psychiatric disorders or chronic physical illness were excluded in this study to mitigate the potential for compounding factors during our analysis. The Extensive Clinical Interview contains items to assess demographics, mental status, and ages at onset for the BPD patients. To avoid the biases due to the low reliability of retrospective evaluation of prodromal symptoms, we defined age at onset as the first reliably-diagnosed hypo/manic or depressive episode according to DSM-IV criteria.

Control subjects were enrolled from hospital staff and students of the School of Medicine in Shanghai that were interviewed by a specialized psychiatrist with SCID-P. Subjects with any psychiatric disorder and chronic physical disease were excluded from our analysis. All subjects were of Han Chinese origin and provided written informed consent before any study-related procedures were performed. This sample has been reported in a previous study

PsyCoLaus MDD sample

Subjects were selected from subjects of European ancestry from a community survey (CoLaus) carried out in the city of Lausanne, Switzerland. Subjects were randomly selected from a complete list of the Lausanne inhabitants aged 35-75 years. All 35 to 66-year old participants were invited by letters also to participate in the psychiatric evaluation (PsyCoLaus). Sixty-seven percent of the participants of the CoLaus study in the age range between 35-66 years accepted the psychiatric evaluation, which resulted in a sample of 3,719 individuals, of whom 92% were of European ancestry. Psychiatric assessment in the PsyCoLaus sub-study included the semi structured Diagnostic Interview for Genetic Studies (DIGS), French version. Cases met DSM-IV criteria for MDD and controls were devoid of any psychiatric disorders. A subset of the 3,419 European subjects who received full psychiatric assessment and gave consent for genetic testing were selected for GWAS genotyping. This research was approved by the local institutional review board. All participants received a detailed description of the goal and funding of the study and signed a written informed consent.

China MDD sample

The China replication MDD sample was from a published GWAS study by Converge consortium.[9] In brief, CONVERGE collected cases of recurrent major depression from 58 provincial mental health centres and psychiatric departments of general medical hospitals in 45 cities and 23 provinces of China. Controls were recruited from patients undergoing minor surgical procedures at general hospitals (37%) or from local community centres (63%). A total of 5,303 Chinese women with recurrent MDD and 5,337 controls without MDD were included in this sample. All subjects were Han Chinese women with four Han Chinese

grandparents. Cases were excluded if they had a pre-existing history of bipolar disorder, psychosis or mental retardation. Cases were aged between 30 and 60 and had two or more episodes of MDD meeting DSM-IV criteria with the first episode occurring between 14 and 50 years of age, and had not abused drugs or alcohol before their first depressive episode. All subjects were interviewed using a computerized assessment system. Interviewers were postgraduate medical students, junior psychiatrists or senior nurses, trained by the CONVERGE team for a minimum of 1 week. The diagnosis of MDD was established with the Composite International Diagnostic Interview (CIDI) (WHO lifetime version 2.1; Chinese version), which used DSM-IV criteria. The interview was originally translated into Mandarin by a team of psychiatrists at Shanghai Mental Health Centre, with the translation reviewed and modified by members of the CONVERGE team.

DNA was extracted from saliva samples using the Oragene protocol. A barcoded library was constructed for each sample. All saliva samples were randomized in allocation to sequencing batches, and experimenters performing the sequencing procedure were blinded to sample allocation and outcome assessment. Sequencing reads obtained from Illumina Hi-seq machines were aligned to Genome Reference Consortium Human Build 37 patch release 5 (GRCh37.p5) with Stampy (v1.0.17) using default parameters after filtering out reads containing adaptor sequences or consisting of more than 50% poor quality (base quality #5) bases. Samtools (v0.1.18) was used to index the alignments in BAMformat, and Picardtools (v1.62) was used to mark PCR duplicates for downstream filtering. The Genome Analysis Toolkit's (GATK, version 2.6) BaseRecalibrator was then run on the BAM files to create base quality score recalibration tables, masking known SNPs and INDELs from dbSNP (version 137, excluding all sites added after version 129). Base quality recalibration (BQSR) was then performed on the BAM files using GATKlite (v2.2.15) while also removing read pairs that did not have the 'properly aligned segment' bit set by Stampy (1–5% of reads per sample).

<u>The Netherlands MDD sample</u>

The Netherlands MDD sample is from a recent Erasmus Rucphen Family (ERF) study,[10] and includes 389 self-reported or clinically diagnosed patients with MDD and 2,056 healthy controls. The ERF study is a cross-sectional cohort including 3,000 living descendants (age range 18-96 years) of 22 couples who lived in the middle of 18th century in an isolated village in the Southwest of the Netherlands and had at least 6 children baptized in the community church. Until the last few decades descendants of these founders have lived in social isolation with minimal immigration (less than 5%). From the year 1848, the population has expanded from 700 up to 20,000 inhabitants.[11] 77% of the fathers and 79% of the mothers in this population have inbreeding coefficient greater than zero. The participants are not selected for any disease or outcome. Detailed information regarding the ERF isolate can be found elsewhere.[11-13] The study protocol of the Netherlands was approved by the medical ethics board of the Erasmus MC Rotterdam, the Netherlands. Written informed consents were provided by all the subjects participating in the study.

Supplementary Table 1. Description of individual replication samples included in this study

Sample	Cases	Case diagnosis	Diagnosis	Interview	Controls	Genotyping	λ
Sweden	1,415	BPD1,BPD2,SAB,BPD-NOS	DSM-IV	/	1,271	Affymetrix 6.0	1.03
Romania	461	BPD1	DSM-IV	SCID-I-P/DIGS	329	Illumina	/
Germany II	181	BPD1,BPD2	DSM-IV	AMDP	527	Illumina	1.05
Australia	330	BPD1,BPD2,SAB,BPD-NOS	DSM-IV	SCID,DIGS	1,811	Illumina	1.00
USA	58	BPD1,BPD2	DSM-IV	DIGS	145	Illumina	/
China-I	198	BPD1,BPD2	DSM-IV	DIGS	135	SNaPShot	/
PsyCoLaus	1,585	MDD	DSM-IV	DIGS	2,362	Illumina	/
China-II	5,303	MDD	DSM-IV	CIDI	5,337	Sequencing	1.07
Netherlands	389	MDD	DSM-IV	CESD	2,056	Illumina	/

Abbreviations:

BPD1, bipolar disorder type 1; BPD2, bipolar disorder type 2; BPD-NOS, bipolar disorder not otherwise specified; SAB, schizoaffective disorder (bipolar type); λ = genomic control lambda; CESD, Center for Epidemiological Studies Depression Scale.

Supplementary Table 2. Demographic data for subjects included in the functional imaging analysis.

	rs9537793			
	**AA (*n* = 77)**	**AG (*n* = 146)**	**GG (*n* = 74)**	***P* value**
**Age (year)**	33.81±10.01	34.01±9.97	32.68±9.35	0.62
**Sex (M/F)**	35/42	73/73	26/48	0.11
**Site (Mannheim/Berlin/Bonn)**	31/17/29	44/42/60	15/24/35	0.12
**Handedness (R/L/both)**	66/9/2	134/8/4	69/4/1	0.43
**Education (year)**	15.38±2.58	15.53±2.24	14.95±2.36	0.21

Supplementary Table 3. Association results of the two risk SNPs in *PCDH17* in each European replication sample

			SNP	rs9563520		rs9537793	
			Position	13:58254630		13:58331407	
			Allele 1/2	T/C		G/A	
	Sample Size		Frequency	0.750/0.250		0.406/0.594	
	Case	Control	Phenotype	*P* value	OR	*P* value	OR
Sweden	1,415	1,271	BPD	0.743	1.024	0.287	1.063
Romania	461	329	BPD	/	/	0.0343	1.256
Germany II	181	527	BPD	0.135	0.808	0.244	0.863
Australia	330	1,811	BPD	0.832	1.024	0.971	1.003
USA	58	145	BPD	0.118	0.665	0.626	1.110
China-I	198	135	BPD	0.689	1.079	0.113	1.298
PsyCoLaus	1,585	2,362	MDD	0.420	1.051	0.310	1.049
China-II	5,303	5,337	MDD	0.952	1.011	0.332	1.030
Netherlands	389	2,056	MDD	0.509	1.073	0.471	1.041
Replications*	9,920	13,973	/	0.323	1.012	0.0170	1.043

Note:
Allele 1 is the effect allele.
OR, odds ratio; BPD, bipolar disorder; MDD, major depressive disorder.
The meta-analysis results in all available replication samples were marked in red.
*In combined replication samples, one-tailed P-value was shown.

Supplementary Table 4. Association results of rs9537793 with educational attainment in two samples.

		**SNP**	**rs9537793**		
		**Position**	13:58331407		
		**Allele 1/2**	G/A		
		**Frequency**	0.406/0.594		
**Sample**	**Phenotype**	**Sample Size**	**OR/Beta**	**SE**	***P* value**
Sample 1[14]	College	95,427	0.970	0.010	**1.04E−03**
	EduYears	101,069	−0.011	0.004	**6.35E−03**
Sample 2[15]	EduYears	293,723	−0.016	0.002	**2.68E−10**

Note:

Allele 1 is the effect allele.

The results showing nominal significance were marked in bold and underlined.

SE, standard error.

The effect size for "College" (binary variable) is shown as OR, and for "EduYears" (quantitative variable) is shown as Beta.

Supplementary Table 5. Association results of rs9537793 with neuroticism

		**SNP**	**rs9537793**		
		**Position**	13:58331407		
		**Allele 1/2**	G/A		
		**Frequency**	0.406/0.594		
**Sample**	**Phenotype**	**Sample Size**	**Beta**	**SE**	***P* value**
Sample 1[16]	Neuroticism	63,661	0.014	0.0056	<u>**1.59E−02**</u>
Sample 2[17]	Neuroticism	170,911	0.010	0.0040	<u>**4.59E−03**</u>

Note:

Allele 1 is the effect allele.

The results showing nominal significance were marked in bold and underlined.

SE, standard error.

Supplementary Table 6. Association results of rs9537793 with subcortical structures[18]

	SNP	rs9537793		
	**Position**	13:58331407		
	**Allele 1/2**	G/A		
	**Frequency**	0.406/0.594		
**Phenotype**	**Sample Size**	**Beta**	**SE**	***P* value**
Amygdala	13,160	−5.924	2.298	**9.95E−03**
Hippocampus	13,163	−9.077	4.578	**4.74E−02**

Note:

Allele 1 is the effect allele.

The results showing nominal significance were marked in bold and underlined.

SE, standard error.

Supplementary Figure 1. Plot of chromosome region showing a genomic area of high linkage disequilibrium with rs9537793 in European populations.

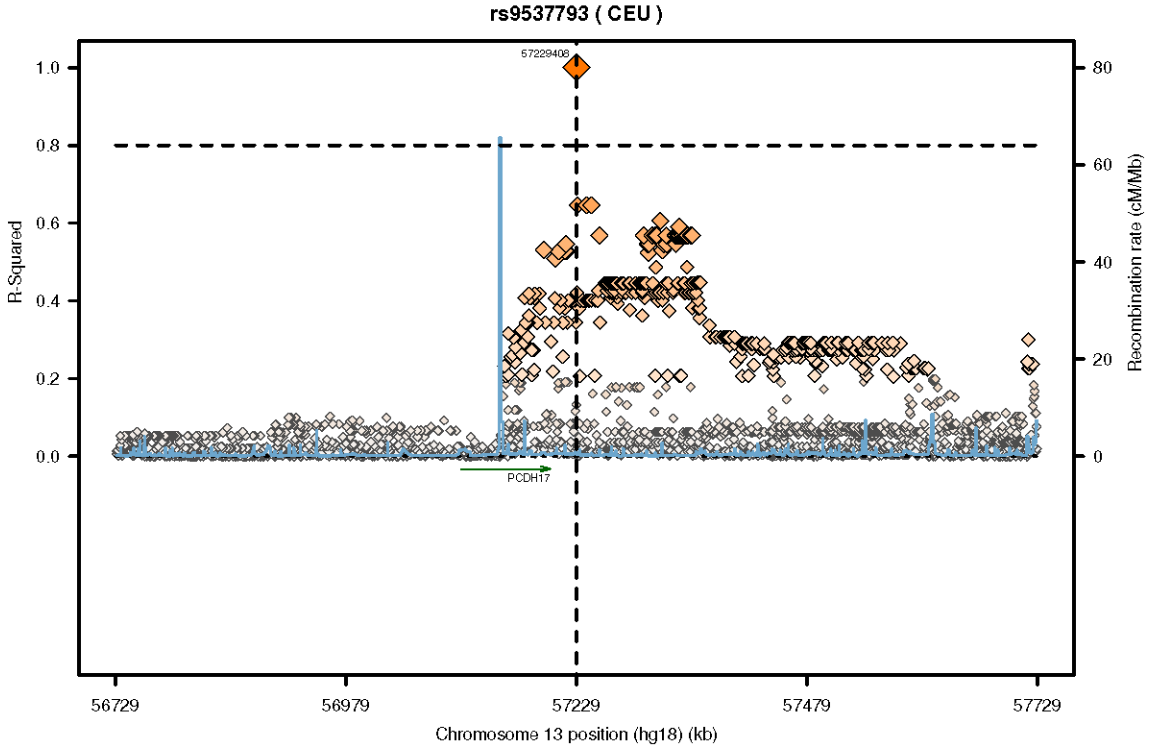

Supplementary Figure 2. Spatial expression profiling of *PCDH17* in human brain tissues from GTEx.[19]

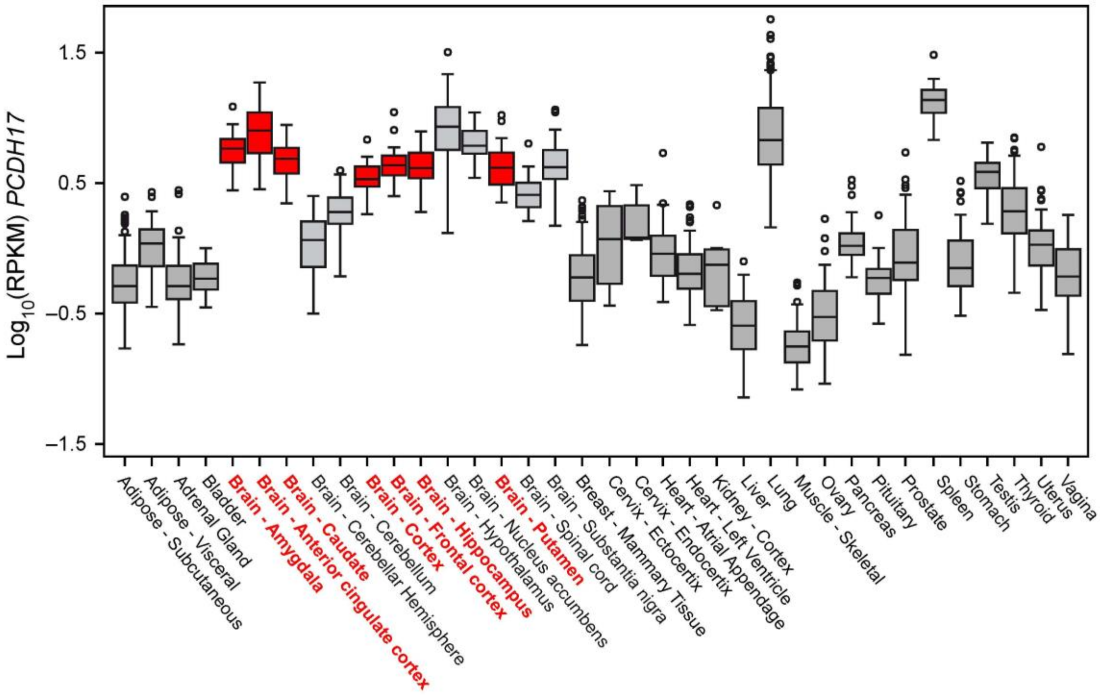

Supplementary Figure 3. ***PCDH17*** **expression was increased in patients with BPD versus with healthy controls (*Post-hoc P* value = 4.69 × 10^{-2}) in a previous thalamic transcriptome study[20] using samples from SMRI neuropathology collection (*n* = 15 each, Schiz./BD/Dep./Controls).**

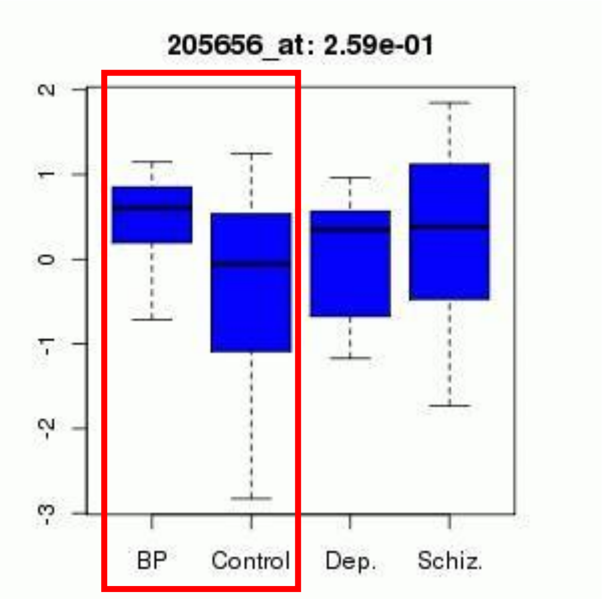

Post-hoc P **value = 4.69 × 10^{-2}**

References

1. Sellgren C, Landen M, Lichtenstein P, Hultman CM, Langstrom N. Validity of bipolar disorder hospital discharge diagnoses: file review and multiple register linkage in Sweden. *Acta Psychiatr Scand* 2011; **124:** 447-453.

2. Vassos E, Steinberg S, Cichon S, Breen G, Sigurdsson E, Andreassen OA *et al.* Replication study and meta-analysis in European samples supports association of the 3p21.1 locus with bipolar disorder. *Biol Psychiatry* 2012; **72:** 645-650.

3. Hammer C, Cichon S, Muhleisen TW, Haenisch B, Degenhardt F, Mattheisen M *et al.* Replication of functional serotonin receptor type 3A and B variants in bipolar affective disorder: a European multicenter study. *Transl Psychiatry* 2012; **2:** e103.

4. Cichon S, Muhleisen TW, Degenhardt FA, Mattheisen M, Miro X, Strohmaier J *et al.* Genome-wide association study identifies genetic variation in neurocan as a susceptibility factor for bipolar disorder. *Am J Hum Genet* 2011; **88:** 372-381.

5. McGuffin P, Farmer A, Harvey I. A polydiagnostic application of operational criteria in studies of psychotic illness. Development and reliability of the OPCRIT system. *Arch Gen Psychiatry* 1991; **48:** 764-770.

6. Schmermund A, Mohlenkamp S, Stang A, Gronemeyer D, Seibel R, Hirche H *et al.* Assessment of clinically silent atherosclerotic disease and established and novel risk factors for predicting myocardial infarction and cardiac death in healthy middle-aged subjects: rationale and design of the Heinz Nixdorf RECALL Study. Risk Factors, Evaluation of Coronary Calcium and Lifestyle. *Am Heart J* 2002; **144:** 212-218.

7. McAuley EZ, Fullerton JM, Blair IP, Donald JA, Mitchell PB, Schofield PR. Association between the serotonin 2A receptor gene and bipolar affective disorder in an Australian cohort. *Psychiatr Genet* 2009; **19:** 244-252.

8. Mitchell PB, Johnston AK, Corry J, Ball JR, Malhi GS. Characteristics of bipolar disorder in an Australian specialist outpatient clinic: comparison across large datasets. *Aust N Z J Psychiatry* 2009; **43:** 109-117.

9. Converge consortium. Sparse whole-genome sequencing identifies two loci for major depressive disorder. *Nature* 2015; **523:** 588-591.

10. Amin N, Belonogova NM, Jovanova O, Brouwer RW, van Rooij JG, van den Hout MC *et al.* Non-synonymous variation in NKPD1 increases depressive symptoms in the European populations. *Biol Psychiatry* 2016.

11. Aulchenko YS, Heutink P, Mackay I, Bertoli-Avella AM, Pullen J, Vaessen N *et al.* Linkage disequilibrium in young genetically isolated Dutch population. *Eur J Hum Genet* 2004; **12:** 527-534.

12. Lopez-Leon S, Choy WC, Aulchenko YS, Claes SJ, Oostra BA, Mackenbach JP *et al.* Genetic factors influence the clustering of depression among individuals with lower socioeconomic status. *PLoS One* 2009; **4:** e5069.

13. Pardo LM, MacKay I, Oostra B, van Duijn CM, Aulchenko YS. The effect of genetic drift in a young genetically isolated population. *Ann Hum Genet* 2005; **69:**

288-295.

14. Rietveld CA, Medland SE, Derringer J, Yang J, Esko T, Martin NW *et al.* GWAS of 126,559 individuals identifies genetic variants associated with educational attainment. *Science* 2013; **340:** 1467-1471.

15. Okbay A, Beauchamp JP, Fontana MA, Lee JJ, Pers TH, Rietveld CA *et al.* Genome-wide association study identifies 74 loci associated with educational attainment. *Nature* 2016; **533:** 539-542.

16. Genetics of Personality Consortium., de Moor MH, van den Berg SM, Verweij KJ, Krueger RF, Luciano M *et al.* Meta-analysis of Genome-wide Association Studies for Neuroticism, and the Polygenic Association With Major Depressive Disorder. *JAMA Psychiatry* 2015; **72:** 642-650.

17. Okbay A, Baselmans BM, De Neve JE, Turley P, Nivard MG, Fontana MA *et al.* Genetic variants associated with subjective well-being, depressive symptoms, and neuroticism identified through genome-wide analyses. *Nat Genet* 2016; **48:** 624-633.

18. Hibar DP, Stein JL, Renteria ME, Arias-Vasquez A, Desrivieres S, Jahanshad N *et al.* Common genetic variants influence human subcortical brain structures. *Nature* 2015; **520:** 224-229.

19. GTEx Consortium. The Genotype-Tissue Expression (GTEx) project. *Nat Genet* 2013; **45:** 580-585.

20. Chu TT, Liu Y, Kemether E. Thalamic transcriptome screening in three psychiatric states. *J Hum Genet* 2009; **54:** 665-675.
